# Expression of MUC17 Is Regulated by HIF1α-Mediated Hypoxic Responses and Requires a Methylation-Free Hypoxia Responsible Element in Pancreatic Cancer

**DOI:** 10.1371/journal.pone.0044108

**Published:** 2012-09-10

**Authors:** Sho Kitamoto, Seiya Yokoyama, Michiyo Higashi, Norishige Yamada, Shyuichiro Matsubara, Sonshin Takao, Surinder K. Batra, Suguru Yonezawa

**Affiliations:** 1 Department of Human Pathology, Field of Oncology, Kagoshima University Graduate School of Medical and Dental Sciences, Kagoshima, Japan; 2 Frontier Science Research Center, Kagoshima University, Kagoshima, Japan; 3 Department of Biochemistry and Molecular Biology, University of Nebraska Medical Center, Omaha, Nebraska, United States of America; University of Kansas Medical Center, United States of America

## Abstract

MUC17 is a type 1 membrane-bound glycoprotein that is mainly expressed in the digestive tract. Recent studies have demonstrated that the aberrant overexpression of MUC17 is correlated with the malignant potential of pancreatic ductal adenocarcinomas (PDACs); however, the exact regulatory mechanism of MUC17 expression has yet to be identified. Here, we provide the first report of the MUC17 regulatory mechanism under hypoxia, an essential feature of the tumor microenvironment and a driving force of cancer progression. Our data revealed that MUC17 was significantly induced by hypoxic stimulation through a hypoxia-inducible factor 1α (HIF1α)-dependent pathway in some pancreatic cancer cells (e.g., AsPC1), whereas other pancreatic cancer cells (e.g., BxPC3) exhibited little response to hypoxia. Interestingly, these low-responsive cells have highly methylated CpG motifs within the hypoxia responsive element (HRE, 5′-RCGTG-3′), a binding site for HIF1α. Thus, we investigated the demethylation effects of CpG at HRE on the hypoxic induction of MUC17. Treatment of low-responsive cells with 5-aza-2′-deoxycytidine followed by additional hypoxic incubation resulted in the restoration of hypoxic MUC17 induction. Furthermore, DNA methylation of HRE in pancreatic tissues from patients with PDACs showed higher hypomethylation status as compared to those from non-cancerous tissues, and hypomethylation was also correlated with MUC17 mRNA expression. Taken together, these findings suggested that the HIF1α-mediated hypoxic signal pathway contributes to MUC17 expression, and DNA methylation of HRE could be a determinant of the hypoxic inducibility of MUC17 in pancreatic cancer cells.

## Introduction

Pancreatic cancer is one of the most aggressive forms of cancer. Because of its aggressive growth and metastatic properties, the overall survival rate of patients with pancreatic cancer is 3–5% [1]. Avascular morphology is a major characteristic of pancreatic cancer, resulting in poor blood and oxygen supply. Consequently, pancreatic cancer contains tumor cells that are at low oxygen tensions, a condition called hypoxia. Accumulating evidence indicates that the hypoxic tumor microenvironment is intimately correlated with solid tumor characteristics including invasion, metastasis, and poor response to anticancer therapies [2], [3]. In this regard, hypoxia-inducible factor 1α (HIF1α) has been identified as a key regulator of the hypoxic response, activating various cancer-related genes such as VEGF, CAIX, and GLUT1.

Mucins are heavily *O*-glycosylated proteins found in the mucus layer, and they are differentially expressed at the cell surface of many epithelia. They are responsible for the physical properties of mucus gels and are involved in epithelial cell protection and maintenance of the local molecular microenvironment [4], [5], [6], [7]. By contrast, there is also increasing evidence that mucins are aberrantly expressed in various cancers, and they have been implicated in malignant transformation as well as tumor cell proliferation, survival, invasion, and metastasis [8], [9], [10]. In this context, recent studies revealed that certain membrane-bound mucins are sufficient for inducing the epithelial-to-mesenchymal transition and thereby represent attractive targets for anticancer treatment [11], [12].

The MUC17 glycoprotein was characterized as a membrane-bound mucin [13]. RNA blot analysis indicated that *MUC17* is mainly expressed in the digestive tract, including the duodenum, ileum, and transverse colon [13], [14]. Under physiological conditions, it has been suggested that endogenous MUC17 plays an important role in cell restitution processes and contributes to colon–mucosal protection [15], [16]. Moniaux et al. reported that MUC17 was aberrantly expressed in pancreatic ductal adenocarcinomas (PDACs) compared with its lack of expression in normal pancreas or pancreatitis [17]. Furthermore, Hirono et al. also reported that MUC17 is an independent prognostic factor associated with lymph node metastasis in PDACs [18]. These findings suggest that MUC17 may have an important role in pancreatic cancer progression. Regarding the regulatory mechanism of MUC17, our previous study suggested that the epigenetic changes, including CpG-DNA methylation and histone modifications at H3-K9 in the MUC17 proximal promoter are key determinants of MUC17 expression [19]. However, it is still unclear what kinds of transcriptional factors are recruited to the MUC17 promoter and involved in the regulation of MUC17 expression.

In the present study, we provide the first evidence describing the regulatory mechanism of MUC17 in the hypoxic tumor microenvironment. Our results show that MUC17 is induced by HIF1α mediating hypoxic response, and the specific DNA methylation determines the hypoxic inducibility of MUC17 in pancreatic cancer cells.

## Materials and Methods

### Ethics statement

Human pancreatic tumor tissues were obtained from Kagoshima University Hospital, and written informed consent was obtained from all study participants. This study was approved by the ethical committee of Kagoshima University Hospital.

### Pancreatic cancer cell lines and human tissue samples

The human pancreatic carcinoma cell lines AsPC1, BxPC3, HPAFII, and PANC1 were purchased from the American Type Culture Collection (Manassas, VA, USA). BxPC3 and AsPC1 cells were cultured in RPMI-1640 medium (Sigma, St. Louis, MO, USA.). HPAFII cells were cultured in Eagle's minimum essential medium (Sigma). PANC1 cells were cultured in D-MEM (Sigma). All media were supplemented with 10% fetal bovine serum (Invitrogen) and 100 U/mL penicillin/100 g/mL streptomycin (Sigma). Hypoxic culture conditions were achieved with a multi-gas incubator containing a gas mixture composed of 94% N_2_, 5% CO_2_, and 1% O_2_ (ASTEC, Japan). All experiments were performed at least three times unless otherwise noted.

### RNA extraction and RT-PCR

Total RNA was extracted using the RNeasy Mini Kit (Qiagen, Valencia, CA, USA) and quantified using a NanoDrop ND-1000 spectrophotometer (NanoDrop, Wilmington, DE, USA). For RT-PCR, the obtained mRNA was reverse-transcribed to cDNA with the High Capacity RNA-to-cDNA Kit (Applied Biosystems, Foster City, CA, USA) according to the manufacturer's instructions. The primers for the subsequent PCR are shown in Table S1. PCR was performed with the AmpliTaq Gold Fast PCR Master Mix (Applied Biosystems) following the manufacturer's protocol.

### Protein extraction and Western blotting

Cells were cultured on 6-cm dishes under hypoxic conditions for their designated times. Total cell lysates were prepared using RIPA buffer containing protease inhibitor cocktail (Nacalai Tesque, Japan). After quantification by the BCA assay (Thermo Scientific), equal amounts was resolved by 3–8% SDS-PAGE and electrotransferred onto PVDF membranes. For MUC17 expression analysis, protein lysates were resolved on 2% agarose gels containing SDS and passively transferred onto PVDF membranes and incubated overnight at room temperature. Membranes were blocked with 1% skim milk/PBST for 2 h and subjected to the standard immunodetection procedure using specific primary antibodies. The primary antibodies were as follows: anti-human MUC17 (rabbit pAb, 1∶1000, generated by one of the authors, Dr. Surinder K. Batra, University of Nebraska Medical Center, Omaha, NE, USA); anti-human HIF1α (mouse mAb, 1∶1000, H1alpha67, Novus Biologicals), and anti-human α-tubulin (mouse mAb, DM1A, Sigma).

### RNA interference

For RNA interference (RNAi) experiments, *HIF1A* expression was silenced using On-Target plus siRNA (Dharmacon) according to the manufacturer's instruction. On-Target plus siControl non-targeting siRNA was used as a control. AsPC1 cells were seeded in 6-cm dishes, and at 50−60% confluency, they were transfected with 13.6 nmol/L siRNA using Lipofectamine RNAiMAX (Invitrogen).

### Dual-Luciferase Reporter Assay

The 5′ flanking sequence (−687 to +53) of human MUC17 was amplified using genomic DNA isolated from AsPC1 cells. The PCR fragment was digested with NheI and XhoI and cloned into the pGL4.17 vector (Promega). To generate HRE mutants of the human MUC17 promoter, a QuikChange Lightning Site-Directed Mutagenesis Kit was utilized (Stratagene). The primer sets are shown in Table S1. For the dual luciferase reporter assay, AsPC1 cells were seeded in a 24-well plate at a density of 1×10^5^ cells/well and transiently transfected with 75 ng of the luciferase reporter plasmid using JetPEI (Polyplus Transfection) according to the manufacturer's protocol. Then, the luciferase activity in the total cell lysates was assayed after 24 h using the Dual-Luciferase Reporter Assay System (Promega) in a Tristar multimode microplate reader LB 941 (Berthold Technologies). The Renilla luciferase reporter gene expressed in a pGL4.74 vector was simultaneously transfected as an internal control.

### Chromatin immunoprecipitation

The chromatin immunoprecipitation (ChIP) assay was performed using a Shearing-ChIP kit and a OneDay ChIP kit (Diagenode, Philadelphia, PA, USA) according to the manufacturer's instructions. In brief, the nucleoprotein complexes were sonicated to reduce the sizes of DNA fragments from 300 to 500 bp by using a Bioruptor (Cosmo Bio). One microgram of normal mouse IgG was used as the negative control, and mouse anti-human HIF1α antibody was used for each immunoprecipitation. Immunoprecipitated DNA was amplified by PCR as described previously. The ChIP primers were designed to target the HRE site of the MUC17 promoter. The primers for the subsequent PCR are shown in Table S1. The PCR conditions were 95°C for 5 min, 34 cycles at 96°C for 5 s, 59°C for 5 s, and 68°C for 7 s, and a final extension reaction at 72°C for 1 min. The amplified products were subjected to 1.0% agarose gel electrophoresis.

### DNA methylation analysis

For CpG demethylation analysis, AsPC1 cells were incubated with 1 µM 5-azadC (Sigma) for 7 days. At the end of the treatment, DNA and protein were extracted for methylation-specific PCR (MSP) and Western blotting. DNA extraction and MSP were performed according to standard methods. In brief, total DNA from cell lines and tissues were extracted using a DNeasy Tissue System (Qiagen). Bisulfite modification of the genomic DNA (2 µg) was performed using an Epitect Bisulfite Kit (Qiagen), and equal amounts of modified DNA were amplified by PCR using the AmpliTaq Gold Fast PCR Master Mix (Applied Biosystems). The specificity of MSP primers is largely influenced by the thermodynamic characteristics of the 3′ end of the proposed primer. Thus, we set the cytidine residue of CpG within HRE as the 3′ end of the MSP primer. The U primer is designed for amplification of sodium bisulfite converted DNA in an unmethylated condition, while the M primer is specific for sodium bisulfite converted methylated DNA. The HRE-targeted primer pairs are shown in Table S1. The PCR conditions were 95°C for 10 min, 40 cycles at 96°C for 5 s, 59°C for 5 s, and 68°C for 5 s, and a final extension reaction at 72°C for 1 min. The amplified products were subjected to 1.0% agarose gel electrophoresis.

To further investigate the correlation of MUC17 expression with the methylation status in biological samples, pancreatic tumor tissues were obtained from the surgically resected pancreatic specimens of 10 patients with PDAC. Normal pancreatic tissues were obtained from the same patients from areas distant from the tumor. All tissue samples were stored at −80°C until use. To conduct MSP and RT-PCR, total DNA and RNA were isolated from the tissues and analyzed as described previously. The bands obtained from MSP and RT-PCR were quantified by Image J soft ware, and the relative level of unmethylation in each sample was calculated as an index of unmethylation status using the equation (%) = U/(U+M). The correlation of MUC17 mRNA expression with the methylation status of HRE was analyzed using Spearman's test.

### Statistical Analysis

In the luciferase reporter and ChIP assays, the statistical differences were determined using a two-sided Student's *t*-test. To investigate the correlation between HRE methylation status and MUC17 expression in tissue samples, Spearman's test was performed using the statistical software R version 2.12.2. P<0.05 was considered significant.

## Results

### Expression of MUC17 is increased under hypoxia in pancreatic cancer cells

**Figure 1:**
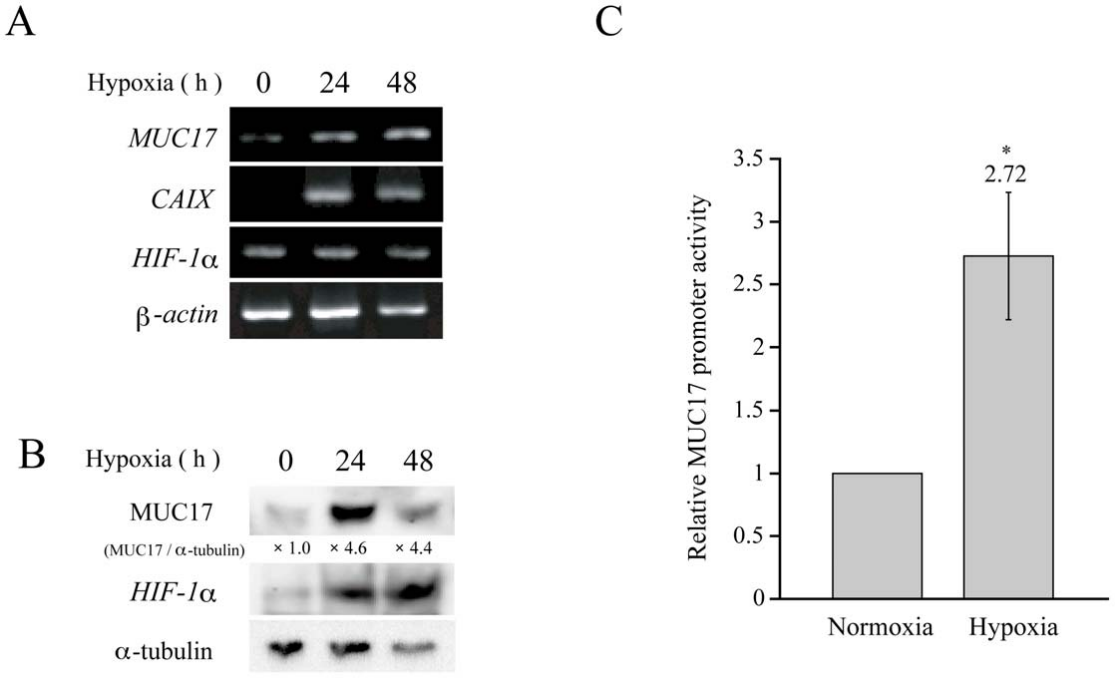
MUC17 expression is enhanced by hypoxia. (A) AsPC1 cells were cultured under hypoxic conditions (1% O_2_) for the indicated times. MUC17 mRNA expression was examined by RT-PCR at each time point. (B) AsPC1 cells were cultured under normoxic or hypoxic conditions for the indicated times. Cell lysates were probed with anti-MUC17, HIF1α, and α-tubulin antibodies by Western blot analysis. The intensities of the bands were quantitated by densitometric scanning, and the ratio of MUC17 to α-tubulin expression is shown under each band as the relative intensity compared with that obtained in normoxic AsPC1 cells. (C) MUC17 promoter activity was measured by a Dual-Luciferase Reporter Assay. After transfection of the MUC17 reporter plasmid, AsPC1 cells were incubated under normoxic or hypoxic conditions for 24 h. Cell lysates were assayed using a luciferase assay kit in a Tristar multimode microplate reader LB941 (Berthold Technologies). Transformation efficiency was normalized on the basis of Renilla luciferase activity. The promoter activity under normoxic conditions was given a value of 1. P values were determined using the Student's *t*-test. * P<0.05.

To examine the effect of hypoxic exposure on MUC17 expression, we cultured human pancreatic cancer cells AsPC1 under hypoxic culture conditions (1% O_2_) for 2 days. In RT-PCR analysis, the expression of MUC17 and CAIX, a hypoxic marker, was significantly increased, whereas the HIF1α mRNA levels were stable under hypoxic conditions, confirming the previously reported posttranscriptional regulation of HIF1α [20]. Western blotting was also employed to examine the MUC17 protein level, and it revealed a prominent induction of MUC17 and HIF1α expression in a time-dependent manner (Figure 1A and 1B). To date, HIF1α is known as a key regulator of hypoxic response in many cancers. HIF1α binds to hypoxia responsive element (HRE, 5′-RCGTG-3′), a binding site for HIF1α, and transactivates many hypoxia-responsive genes including VEGF, GLUT1, and CAIX. We conducted sequence analysis of the approximately 3.0-kb MUC17 promoter with MatInspector software (Genomatix) and found a putative HRE (+37/+41) in the proximal promoter region of MUC17. Thus, we hypothesized that MUC17 overexpression under hypoxic conditions is mediated by HIF1α. To examine the effect of hypoxia on the transcriptional activity of the MUC17 promoter, we constructed an MUC17 promoter–luciferase reporter vector containing the HRE site. Under hypoxic conditions, AsPC1 cells transfected with this construct exhibited significantly increased reporter activity (Figure 1C), suggesting that the enhancement of MUC17 expression under hypoxic conditions is due to an increase in transcriptional activity.

### Hypoxic induction of MUC17 is regulated by the HIF1α-dependent signal pathway

**Figure 2:**
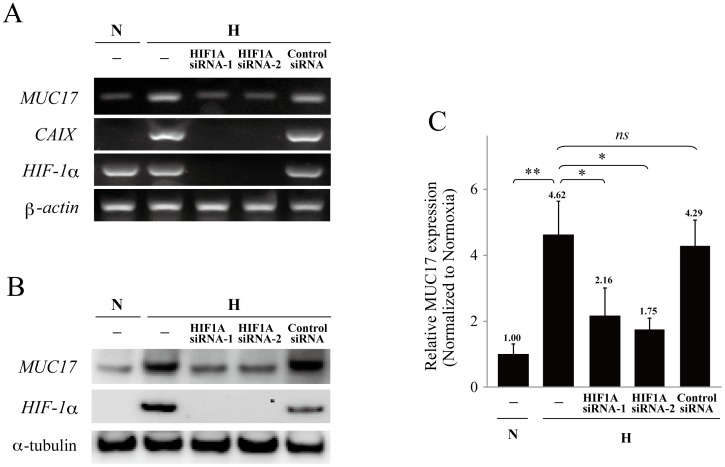
Hypoxic induction of MUC17 is dependent on HIF1α in AsPC1 cells. (A) After the transfection of HIF1A siRNAs, AsPC1 cells were cultured under normoxia (N) or hypoxia (H) for 24 h. The level of mRNA was measured by RT-PCR. (B) Cell lysates from AsPC1 cells treated with HIF1A siRNAs were immunoblotted with the indicated antibodies. α-tubulin served as a loading control. (C) The densities of the acquired bands from Western blotting analysis were quantified and expressed as relative fold increases compared with that obtained from mock cells under hypoxic culture conditions. ns, not significant. * P<0.05, ** P<0.005.

In order to investigate whether HIF1α is really involved in the hypoxic induction of MUC17, we examined the effect of HIF1α silencing on the hypoxic MUC17 induction in AsPC1 cells by using siRNAs. Under hypoxia, control siRNA had no effect on transcription of MUC17, HIF1α, CAIX, and ß-actin. On the contrary, treatment with siRNAs targeting HIF1α led to a prominent decrease of hypoxic MUC17 induction as well as CAIX (Figure 2B). The same result was observed at the protein level (Figure 2B), indicating the involvement of HIF1α in hypoxic MUC17 induction.

### Hypoxia enhances the recruitment of HIF1α to HRE and activates MUC17 transcription

**Figure 3:**
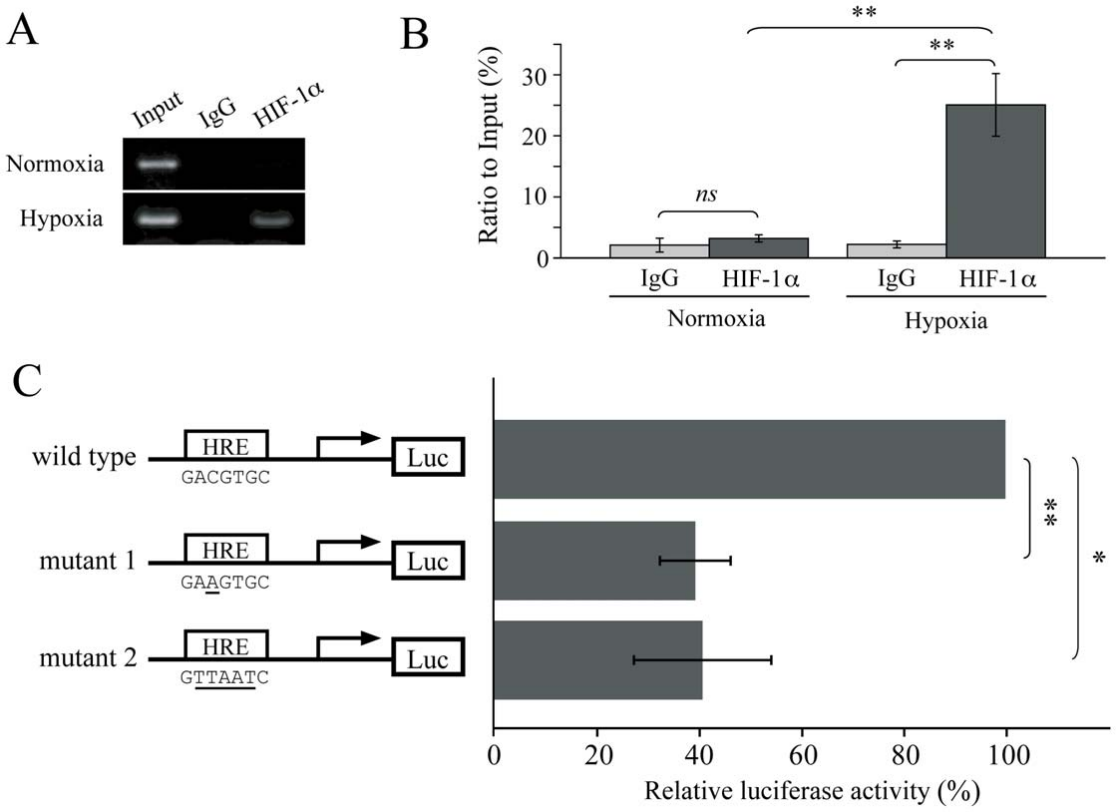
Hypoxia enhances the recruitment of HIF1α to HRE and activates MUC17 transcription. (A) Binding of HIF1α to chromatin was confirmed by a ChIP assay. AsPC1 cells were cultured under normoxia or hypoxia for 24 h. PCR was performed with specific primers covering HRE. (B) The densities of the acquired bands in panel (A) were quantified using Image J (NIH) and normalized to Input included in each experiment. (C) To evaluate the transactivation activity of HIF1α through HRE, a dual luciferase assay was conducted. AsPC1 cells were transfected with wild-type or HRE mutant MUC17 promoter constructs under hypoxic conditions for 24 h. P values were determined using Student's *t*-test. ns, not significant. * P<0.01, ** P<0.001.

To assess the involvement of HIF1α in MUC17 transcriptional regulation, we performed ChIP assays. The result revealed that the anti-HIF1α antibody significantly enriched the MUC17 promoter fragments harboring HRE under hypoxic conditions but not under normoxic conditions (Figure 3A and 3B). Further, we tested whether HRE was indeed essential for hypoxia-inducing MUC17 transactivation. Reporter vectors harboring mutations at the HRE site were constructed and transfected into AsPC1 cells under hypoxic conditions. As shown in Figure 3C, cells transfected with either mutant vectors exhibited significantly reduced transactivation compared with that of the wild-type promoter construct-transfected cells, indicating the essential role of the HRE site in the hypoxic MUC17 induction.

### Differential expression pattern of MUC17 induced by hypoxia in various pancreatic cancer cell lines

**Figure 4:**
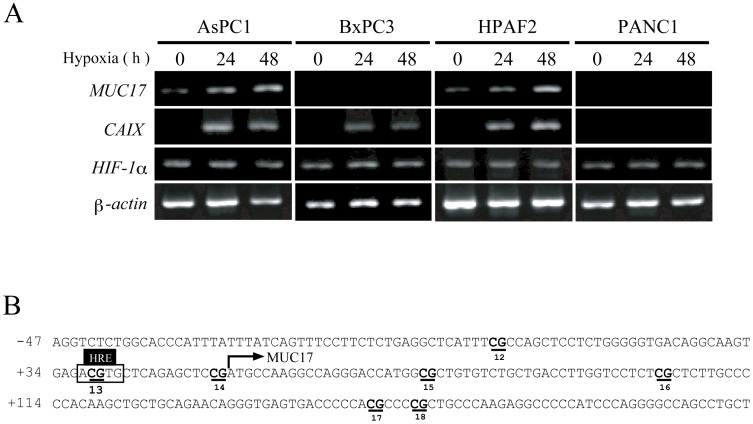
Methylation status of HRE within the MUC17 proximal promoter region. (A) Differential expression pattern of MUC17 induced by hypoxic exposure in pancreatic cancer cell lines. Each pancreatic cell line was cultured under normoxic (N) or hypoxic (H) conditions. Level of mRNA was determined by RT-PCR at each time point. (B) The human MUC17 gene promoter sequence, which spans positions −687 to +302 with respect to the transcription start site. The numbers of the CpG sites are underlined. The HRE site contains the CpG site (No. 13).

Furthermore, we evaluated whether the hypoxic induction of MUC17 is a universal event in pancreatic cancer cells. Additional cell lines (BxPC3, HPAFII, and PANC1) were cultured under hypoxic conditions for 2 days, and mRNA expression levels were monitored. The results revealed that MUC17 expression levels in AsPC1 and HPAFII cells were significantly increased under hypoxic conditions. In contrast, only a faint increase was observed in BxPC3 and PANC1 cells (Figure 4A). In this context, we recently reported that DNA hypomethylation is a key factor for MUC17 expression in pancreatic cancer (Figure 4B). Interestingly, AsPC1 and HPAFII cells, which exhibited prominent MUC17 induction under hypoxia, displayed an almost completely unmethylated HRE (No. 13) within the MUC17 promoter, whereas BxPC3 and PANC1 cells had highly methylated HREs [19]. Thus, we hypothesized that the DNA methylation status of HRE determines MUC17 induction in response to hypoxia.

### DNA methylation status of HRE determined the hypoxia inducibility of MUC17 expression

**Figure 5:**
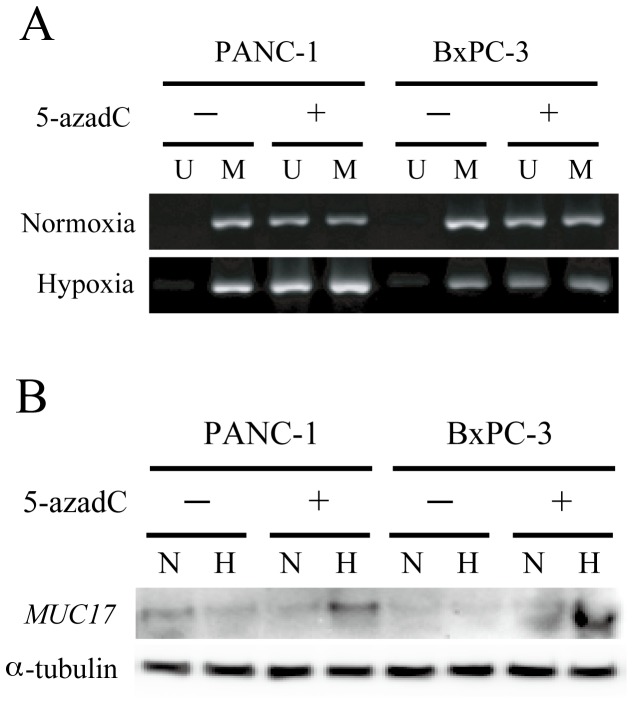
Methylation status of the HRE site determines the sensitivity of MUC17 to HIF1α-induced transactivation. (A) Demethylation of CpG sites in the MUC17 promoter in BxPC3 and PANC1 cells after 1 µM 5-azadC treatment. MUC17-negative/low cell lines were treated with or without 5-azadC for 7 days. The methylation status of the MUC17 promoter harboring HRE was examined by MSP. The PCR products labeled M (methylated) were amplified by methylation-specific primers, and those labeled U (unmethylated) were amplified by primers specific for unmethylated DNA. (B) Restoration of MUC17 expression in pancreatic cancer cell lines by 5-azadC treatment. Cells were treated with or without 5-azadC for 7 days. During the last 24 h, each group was cultured under normoxic (N) or hypoxic (H) conditions. MUC17 expression was examined by Western blotting.

To investigate the involvement of DNA methylation of HRE in MUC17 expression under hypoxic conditions, we treated BxPC3 and PANC1 cells with 5-AzadC (a DNA demethylating agent) for 7 days, and the cells were incubated for another 24 h under normoxia or hypoxia. First, we confirmed the demethylation effect of 5-AzadC treatment on HRE by MSP (Figure 5A). Then, we examined the MUC17 expression in cells treated with 5-AzadC under hypoxic conditions, resulting in the restoration of MUC17 expression only in cells treated 5-AzadC treatment under hypoxia (Figure 5B).

### MUC17 expression correlates with hypomethylation at HRE within the promoter region

**Figure 6:**
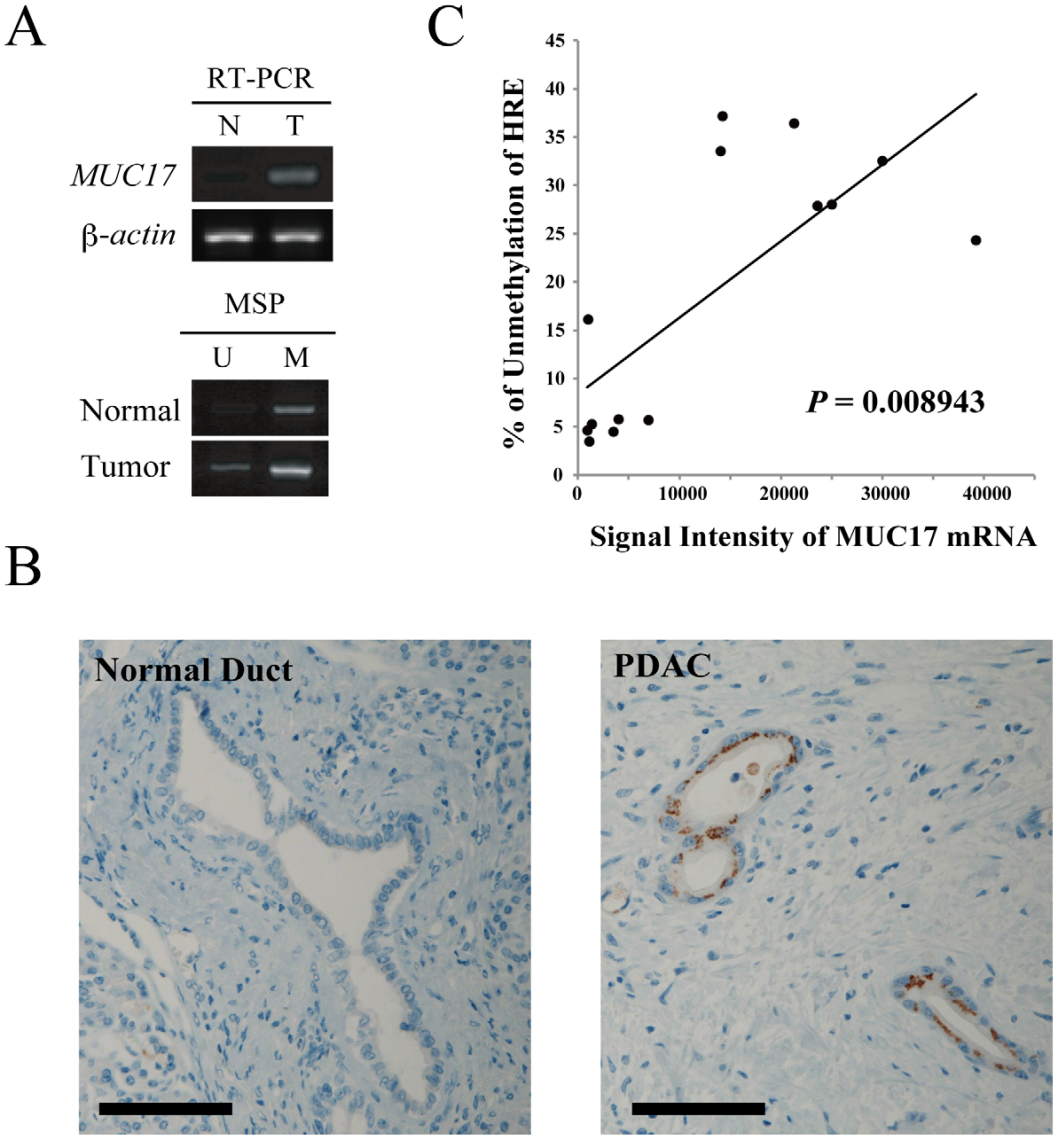
MUC17 expression is correlated with the hypomethylation of HRE within the MUC17 promoter in tissues from patients with PDAC. (A) MUC17 mRNA expression and the HRE methylation status in the normal pancreas (N) and pancreatic tumor tissues (T) was examined by RT-PCR and MSP, respectively. (B) Representative immunohistochemical staining data for MUC17 in a patient with PDAC. Scale bar, 100 µm. (C) Correlation of MUC17 mRNA expression and the HRE methylation status was analyzed by Spearman's test. The densities of the acquired bands were quantified using Image J, and the relative amount of unmethylation in each sample was calculated as an index of the aberrant unmethylation status using the equation (%) = U/(U+M).

Furthermore, to evaluate the biological significance of HRE methylation in MUC17 expression, we examined MUC17 expression and methylation status in normal and pancreatic tumor tissues from patients with PDACs by RT-PCR and MSP, respectively. Consistent with the previous reports [17], [18], MUC17 mRNA and protein are overexpressed in PDAC (Figure 6A and B). In the MSP analysis using 10 paired PDAC samples (non-cancerous tissues; n = 10, cancerous tissues; n = 10), unmethylated signals were significantly observed in tissues with PDAC as compared to those from non-cancerous tissues (Two-tailed Student's *t*-test, P = 0.007). We also investigated the correlation between MUC17 mRNA expression and the methylation status of HRE. The level of MUC17 mRNA was strongly correlated to the hypomethylation status of HRE within the MUC17 promoter in pancreatic cancer (Figure 6C).

## Discussion

Recent studies have identified human MUC17, which is overexpressed in PDACs, as an independent prognostic factor of lymph node metastasis [17], [18]. Although little is known about the functional role of MUC17 in cancer, understanding the regulatory mechanism of MUC17 can be a key step in developing new strategies for cancer diagnosis and treatment.

In the present study, we investigated the hypoxic regulation of MUC17 in pancreatic cancer cells. Our results demonstrated that MUC17 is significantly induced by hypoxia in a time-dependent manner, and this upregulation is directly mediated by HIF1α. Moreover, the use of four cell lines with different MUC17 hypoxic inducibilities allowed us to propose a model for the epigenetic regulation of MUC17 in pancreatic cancer. We propose that DNA methylation of HRE inhibits HIF1α-mediated MUC17 transactivation in cells with low MUC17 hypoxic inducibility, whereas progressive hypomethylation of HRE allows a prominent increase in MUC17 expression under hypoxic conditions in cells with high hypoxic inducibility. There is increasing evidence that the methylation of CpG sites in promoter regions affects the induction of gene expression by HIF1α, presumably by limiting the access of transcriptional factors to *cis*-acting elements [21], [22]. As shown in Figure 6, hypomethylation of HRE within the MUC17 promoter is a frequent event in the pancreatic tissues of patients with PDAC. Although further studies are needed to elucidate the other factors involved in MUC17 expression, this could explain why MUC17 is overexpressed in pancreatic cancer. Regarding the lack of CAIX induction in PANC1 cells, it has been demonstrated that CAIX expression is, at least in part, regulated by site-specific CpG hypomethylation at −74 bp in the promoter in renal cell carcinoma and other cancer cell types [23], [24], [25]. Therefore, this response may also be affected by the DNA methylation status of HRE within the CAIX promoter.

There are some implications for considering the functional role of MUC17 in cancer development. In solid tumors, because of the unrestricted proliferation of cancer cells and inadequate vascularization, oxygen starvation (hypoxia) and nutrient deprivation are recognized as essential features of the tumor microenvironment and a driving force of cancer progression. Recently, it has been reported that MUC1, which is also identified as a hypoxia-inducible mucin, could promote autophagy, providing a survival advantage in the low glucose-stressed microenvironment through the suppression of glucose deprivation-induced increases of ROS levels in colon cancer [26]. In addition, it also has been reported that stable transfection of small hairpin RNA targeting endogenous MUC17 in colon cancer LS174T cells resulted in reduced cell aggregation, reduced cell–cell adherence and migration, and increased susceptibility to apoptosis [15]. These findings indicate that MUC17 might have some functions, including cell migration, invasion, resistance to apoptosis and adaptation to stressed microenvironments in pancreatic cancer progression.

Alterations of DNA methylation are one of the most remarkable epigenetic changes in human cancer. The accumulating evidence suggests that aberrant DNA methylation is frequently observed even in the early and precancerous stages of human carcinogenesis and it may be an indicator of carcinogenetic risk, an early diagnostic marker for cancer, and a biological predictor of cancer malignancies [27], [28]. It has been reported that epigenetic modifications including DNA methylation largely contribute to the expression of human mucin family genes in cancer [19], [29], [30], [31], [32], [33], [34]. Zhu et al. have reported an aberrant increase in both the expression and hypomethylation of MUC4 during PanIN-PDAC progression [35]. In addition, we developed a novel method for detecting DNA methylation patterns called MSE, which enabled the detection of altered methylation in pancreatic juice and revealed the significantly different methylation patterns in the MUC1 promoter between intraductal papillary mucinous neoplasm and PDAC, indicating its potential diagnostic use [36]. Although further studies are needed to clarify the clinicopathological importance and precise mechanisms of MUC17 expression, these findings raise the possibility that the methylation status of the MUC17 promoter is an epigenetic marker for the diagnosis of carcinogenic risk and the prediction of outcomes of patients with PDAC. Taken together, our study demonstrates for the first time that MUC17 expression is enhanced by the HIF1α-mediated hypoxic response and that DNA methylation of HRE is a key determinant of the hypoxic inducibility of MUC17 in pancreatic cancer. In the future, the significance of these findings in pancreatic cancer pathogenesis will be explored.

## Supporting Information

Table S1 Synthetic oligonucleotides used in this study. Synthetic oligonucleotides listed with the position number with respect to the transcriptional start site. In MSP analysis, * indicates the U primer for unmethylated alleles. ** indicates the M primer for methylated alleles.
(DOC)
