# Supplementary material for: Expression of MUC17 Is Regulated by HIF1α-Mediated Hypoxic Responses and Requires a Methylation-Free Hypoxia Responsible Element in Pancreatic Cancer
Source: PLoS One. 2012 Sep 10;7(9):e44108. doi: 10.1371/journal.pone.0044108 (PMC3438193; doi:10.1371/journal.pone.0044108)
Supplement: Table S1 — Synthetic oligonucleotides used in this study. Synthetic oligonucleotides listed with the position number with respect to the transcriptional start site. In MSP analysis, * indicates the U primer for unmethylated alleles. ** indicates the M primer for methylated alleles. (DOC) [file pone.0044108.s001.doc]

**Table S1. Synthetic oligonucleotides used in this study**

Name Primer sequence Position

semi-quantitative PCR primers

MUC17-F2 GCTGTGTCTGCTGACCTTGG exon 1

MUC17-R2 TGGCACTGACGGTTCAAGAC exon 2

CAIX-F1 CGGAAGAAAACAGTGCCTATGAG exon 6

CAIX -R1 CAGGGCGGTGTAGTCAGAGA exon 7

HIF1A-F1 CTCAAAGTCGGACAGCCTCA exon 14

HIF1A-R1 CCCTGCAGTAGGTTTCTGCT exon 15

ACTB-F1 CTCTTCCAGCCTTCCTTCCTG exon 4

ACTB-R1 GAAGCATTTGCGGTGGACGAT exon 6

MUC17 promoter primers

MUC17-PF1 GCTAGCGAGACAGAGTCTTGCTCTGTTGC −687 to −665

MUC17-PR1 CTCGAGCGGAGCTCTGAGCACGTCTC +34 to +53

Mutagenesis oligonucleotides

M17mt1-a GACAGGCAAGTGAGAAGTGCTCAGAGCTCCG GACGTGC → GAAGTGC

M17mt1-as CGGAGCTCTGAGCACTTCTCACTTGCCTGTC GACGTGC → GAAGTGC

M17mt2-a GGGGTGACAGGCAAGTGAGTTAATCTCAGAGCTCCGCTCGAGG GACGTGC → GTTAATC

M17mt2-as CCTCGAGCGGAGCTCTGAGATTAACTCACTTGCCTGTCACCCC GACGTGC → GTTAATC

MSP primers

MUC17-UF* GTTGATAATTTTTATGTTTATGGGTTGTT −207 to −179

MUC17-UR* CTAACCTTAACATCAAAACTCTAAACACA +38 to +66

MUC17-MF** GTTGATAATTTTTATGTTTATGGGTTGTC −207 to −179

MUC17-MR** CCTTAACATCGAAACTCTAAACACG +38 to +62

ChIP primers

MUC17-CF1 CCAGTGTCTCAGGGACCAAAG −66 to −46

MUC17-CR1 CCAAGGTCAGCAGACACAGC +77 to +96

Synthetic oligonucleotides listed with the position number with respect to the transcriptional start site. In MSP analysis, * indicates the U primer for unmethylated alleles. ** indicates the M primer for methylated alleles.
